# A Full Good Manufacturing Practice–Compliant Protocol for Corneal Stromal Stem Cell Cultivation

**DOI:** 10.21769/BioProtoc.5074

**Published:** 2024-09-20

**Authors:** Mithun Santra, Yen-Michael S. Hsu, Shaheen Khadem, Sydney Radencic, Martha L. Funderburgh, Onkar B. Sawant, Deepinder K. Dhaliwal, Vishal Jhanji, Gary H.F. Yam

**Affiliations:** 1 Corneal Regeneration Laboratory, Department of Ophthalmology, University of Pittsburgh, Pittsburgh, PA, USA; 2 Immunologic Monitoring and Cellular Products Laboratory, Hillman Cancer Centre, University of Pittsburgh, Pittsburgh, PA, USA; 3 Center for Vision and Eye Banking Research, Eversight, Cleveland, OH, USA; 4 McGowan Institute for Regenerative Medicine, University of Pittsburgh, Pittsburgh, PA, USA; 5 Center for Cell and Gene Therapy, Gift of Life Marrow Registry, Boca Raton, FL, USA

**Keywords:** Corneal opacities, Corneal stromal stem cells, Anterior limbal stroma, Good manufacturing practices, Standard operating protocol

## Abstract

Corneal scarring, a significant cause of global blindness, results from various insults, including trauma, infections, and genetic disorders. The conventional treatment to replace scarred corneal tissues includes partial or full-thickness corneal transplantation using healthy donor corneas. However, only 1 in 70 individuals with treatable corneal scarring can undergo surgery, due to the limited supply of transplantable donor tissue. Our research focuses on cell-based strategies, specifically ex vivo–expanded corneal stromal stem cells (CSSCs), to address corneal scarring. Preclinical studies have demonstrated the efficacy of CSSC treatment in reducing corneal inflammation and fibrosis, inhibiting scar formation, and regenerating native stromal tissue. Mechanisms include CSSC differentiation into stromal keratocytes and the expression of regenerative cytokines. Here, we present a good manufacturing practice (GMP)-compliant protocol to isolate and expand human CSSCs. This method paves the way to produce clinical-grade CSSCs for transplantation and clinical trials.

Key features

• This protocol utilizes surgical skills to dissect human corneal tissues for CSSC isolation.

• The yield and features of CSSCs rely on donor tissue quality (freshness) and have donor-to-donor variability.

• Up to 0.5 billion CSSCs can be generated from a single cornea specimen, and cells at passage 3 are suitable for treatment uses.

## Graphical overview


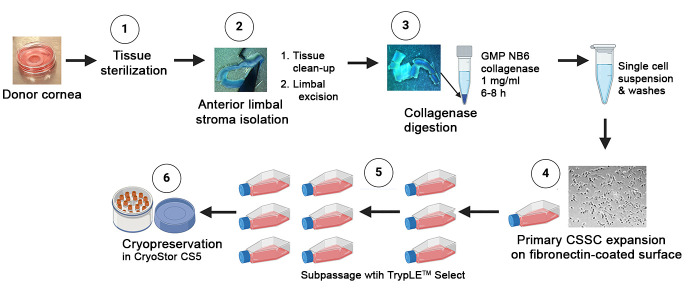


## Background

Damage to the cornea, caused by physical trauma, chemical burns, infections such as microbial or viral keratitis, or genetic disorders like corneal dystrophies and keratoconus, initiates a complex cascade of inflammatory and fibrotic reactions [1,2]. These events lead to tissue damage and remodeling, which involves the generation of stromal fibroblasts and myofibroblasts. These cells produce a repair-type extracellular matrix (ECM) to heal the wound. However, excessive deposition of ECM, including type-III collagen and fibronectin extradomain A (EDA-Fn), leads to the formation of haze and opacities, eventually resulting in corneal scarring that can compromise the corneal clarity and integrity, causing visual impairment [3]. While superficial scarring can often heal within months, deep or dense scars may develop and cause permanent vision loss.

Corneal scarring is the fourth leading cause of global blindness, affecting approximately 2 million people [4]. Each year, over 350,000 children are affected by this condition [5,6]. This is a major concern, as it disproportionately affects younger individuals and low-income rural communities due to factors such as communicable diseases, higher risk of injury, and limited access to treatment options [7]. Various options are available to treat scarred corneas, including pharmacological treatments such as steroids, immune modulation, and grafting with donor corneal materials [8]. Pharmacological therapies offer moderate correction for corneal haze and mild opacities. Corneal transplantation, such as penetrating and lamellar keratoplasty, is the standard for moderate-to-severe scarring and opacities occurring along the visual axis. However, the limited global supply of transplantable donor corneas, immune response (depending on the histocompatibility context of the host that could affect the long-term graft survival), risk of graft rejection, and potential surgical complications restrict the widespread use of keratoplasty [7,9].

Our research team investigated the use of human corneal stromal stem cells (CSSCs) to reduce corneal inflammation and fibrosis; the treatment prevented corneal scarring and restored corneal clarity in pre-clinical models of stromal injury created by mechanical debridement, alkali burns, or liquid nitrogen [10–17]. Our team follows a specific set of protocols to isolate primary donor CSSCs through microdissection of the anterior limbal stroma and then culture them using a stem cell growth medium called JM-H [10,18,19]. These CSSCs can be propagated up to 50–70 doublings before undergoing senescence and changes in cell features [20]. They express stem cell markers like ATP-binding cassette transporter superfamily G member 2 [ABCG2], nestin, Pax6, and Bmi-1, undergo stromal keratocyte differentiation, express anti-inflammatory tumor necrosis factor-stimulating gene 6 [TSG-6] and transforming growth factor b3 [TGFb3], and anti-fibrosis microRNAs (hsa-miR-29a and 381) [10,12,14,17,18].

In order to apply these cells in clinical settings, it is necessary to generate them using a good manufacturing practice (GMP)-compliant protocol. Regulatory approval for cell therapy is challenging, with only a small number gaining approval in comparison to pharmaceutical drugs [21]. Quality, safety, and effectiveness are extremely important for clinical trials, guided by good clinical practice principles [22,23]. To ensure regulatory compliance and consistent results, a quality management system that encompasses quality assessment and quality control is crucial for cell therapy [23]. We have developed GMP-compliant protocols for clinical-grade CSSC production, which laid the groundwork for human testing in clinical trials.

## Materials and reagents

**Biological materials**

1. Donor corneas

**GMP Reagents**
1. Dulbecco’s modified Eagle medium (DMEM/F12) (Thermo Fisher Scientific, Gibco, catalog number: 12634-010)
2. Phosphate buffered saline (PBS) (Thermo Fisher Scientific, Gibco, catalog number: 10010-023)
3. DMEM (1 g/L D-glucose, with GlutaMAX) (Thermo Fisher Scientific, Gibco, catalog number: 10567-014)
4. Ham’s F12 (Thermo Fisher Scientific, Gibco, catalog number: 31765-035)
5. BioWhittaker antibiotic-antimycotic (100×) (Lonza, catalog number: 17-745E)
6. Insulin-transferrin-selenium (100×) (Thermo Fisher Scientific, Gibco, catalog number: 41400-045)
7. Flexbumin (25%) (BioSupply, catalog number: 00944-0493-01)
8. 2-Phospho-L-Ascorbate (Tocris Biosci, catalog number: 5778)
9. Recombinant human epidermal growth factor (EGF) (Thermo Fisher Scientific, Gibco, catalog number: PHG6045)
10. Recombinant human platelet-derived growth factor-BB (PDGF-BB) (R&D, catalog number: 220-GMP-050)
11. Dexamethasone (Dex) (USP micronized) (Spectrum, catalog number: DE121)
12. Human serum (Innovative Res, catalog number: ISER-36670)
13. Collagenase (Collagenase NB6) powder (Nordmark, catalog number: N0002779)
14. Fibronectin coating substrate (MedChemExpress, catalog number: HY-P70593G)
15. TrypLE Select CTS (Thermo Fisher Scientific, Gibco, catalog number: A12859-01)
16. CryoStor CS10 (serum-free DMSO 10%) (BioLife Solutions, catalog number: 210373)
17. Trypan blue (0.4%) (Invitrogen, catalog number: T10282)
18. Hydrochloric acid (HCl) (Sigma-Aldrich, catalog number: H9892)

**Solutions**
1. Recombinant human EGF (see Recipes)
2. Recombinant human PDGF (see Recipes)
3. Dexamethasone (Dex) solution (see Recipes)
4. Collagenase NB6 solution (see Recipes)
5. GMP fibronectin (see Recipes)
6. CryoStor CS5 (see Recipes)
7. GMP stem cell culture medium (see Recipes)

**Recipes**
1. **Recombinant human EGF**
  Store the lyophilized EGF at -20 °C under desiccation until reconstitution. Prior to opening, briefly centrifuge the vial of lyophilized EGF at 400× *g* for 10–15 s to bring the contents to the bottom. Reconstitute EGF powders (500 mg) with sterile distilled water (1 mL volume) to a concentration of 0.5 mg/mL. Further dilute to 10 mg/mL with sterile DMEM/F12 added with 0.1% Flexbumin (carrier protein), aliquot at 0.5 mL, and store at -80 °C until use or at 2–8 °C for one month.
  **Table:** 

Reagent	Final concentration	Quantity
Recombinant human EGF	10 mg/mL	100 mL from 0.5 mg/mL stock
DMEM/F12	1×	5 mL
Flexbumin	0.1% (v/v)	20 mL from 25% stock
2. **Recombinant human PDGF**
  Store the lyophilized PDGF-BB at -20 °C under desiccation until reconstitution. Prior to opening, briefly centrifuge the vial of lyophilized PDGF-BB at 400× *g* for 10–15 s to bring the contents to the bottom. Reconstitute PDGF-BB powders (50 mg) with sterile hydrochloric acid (HCl, 4 mM) with 0.1% Flexbumin (carrier protein) to a concentration of 10 mg/mL, aliquot at 0.5 mL, and store at -80 °C until use or at 2–8 °C for one month.
  **Table:** 

Reagent	Final concentration	Quantity
Recombinant human PDGF-BB	10 mg/mL	One vial of 50 mg
HCl	4 mM	5 mL
Flexbumin	0.1% (v/v)	20 mL from 25% stock
3. **Dexamethasone (Dex) solution**
  Store the lyophilized Dex at -20 °C under desiccation until reconstitution. Prior to chemical preparation, wear PPE and a suitable eye protection device as Dex has a risk of causing eye damage/irritation. Thaw Dex at room temperature for 10–20 min. Weigh approximately 50 mg of powder and calculate the amount of distilled water to make a stock concentration of 10 mM with reference to the molecular weight 392.47. Further dilute to 10 mM with sterile DMEM/F12, aliquot at 100 mL volume, and store at -80 °C until use or at 2–8 °C for one month.
  **Table:** 

Reagent	Final concentration	Quantity
Dexamethasone	10 mM	5 mL from 10 mM stock
DMEM/F12	1×	5 mL
4. **Collagenase NB6 solution**
  Store the lyophilized collagenase powder at 2–8 °C under desiccated conditions. Dissolve 100 mg of powder with 10 mL of sterile PBS (cGMP) to make a stock concentration of 10 mg/mL. Aliquot the solution to 0.15 mL in sterile 2 mL Eppendorf tubes and store at -20 °C. Prior to tissue digestion, thaw the collagenase aliquot at room temperature for 10–15 min and add 1.35 mL of DMEM/F12 with 0.1% Flexbumin and 1× antibiotics/antimycotic to make a working concentration of 1 mg/mL.
  **Table:** 

Reagent	Final concentration	Quantity
Collagenase NB6 powder	1 mg/mL	0.15 mL of 10 mg/mL stock
DMEM/F12	1×	1.35 mL
Flexbumin	0.1% (v/v)	6 mL
Antibiotics/antimycotic	1×	15 mL from 100× stock
5. **GMP fibronectin**
  Store lyophilized fibronectin at -20 °C under desiccation for a maximum of 2 years. Thaw the powder at room temperature for 10–20 min. Prior to opening, briefly centrifuge the vial at 400× *g* for 10–15 s to bring the contents to the bottom. Add 1 mL of injection water to 500 mg of fibronectin powder to make a stock concentration of 500 mg/mL, aliquot to 50 mL volume, and store at -80 °C for up to a year or at 2–8 °C for a week.
**Fibronectin coating:** Dilute GMP fibronectin at 500 mg/mL stock with sterile PBS to 10 mg/mL working concentration. Apply 1 mL per TCF25 (tissue culture flask, 25 cm^2^) and coat at room temperature for 15–30 min. Remove the solution, and the surface is ready for cell seeding.
  **Table:** 

Reagent	Final concentration	Quantity
Fibronectin	10 mg/mL	20 mL of 500 mg/mL stock
PBS	1×	1 mL
6. **CryoStor CS5**
  Store CryoStor CS10 at 2–8 °C. Prior to use for CSSC cryopreservation, dilute CS10 with GMP culture medium (1:1 v/v) to make a working CS5 solution with 5% DMSO and keep at room temperature for immediate cell storage or at 2–8 °C for one week. The step of cell freezing requires the use of a controlled temperature apparatus (e.g., Mr. Frosty^TM^ freezing container with isopropanol).
  **Table:** 

Reagent	Final concentration	Quantity
GMP culture medium	0.5×	0.5 mL
CryoStor CS10	0.5×	0.5 mL
7. **GMP stem cell culture medium (GMP SCCM)**
  Thaw the frozen reagents (including recombinant human EGF, recombinant human PDGF-BB, and Dex) at room temperature for approximately 10–15 min or at 2–8 °C overnight. Mix the thawed solutions by gently inverting the Eppendorf vial a few times, followed by a brief spinning with a benchtop micro-centrifuge.
  To prepare SCCM with 500 mL volume, mix 300 mL of DMEM (1,000 mg/L D-glucose) and 200 mL of Ham’s F12. Add reagents with the required volume, filter-sterilize, and bring the culture medium to room temperature before use.
  **Table:** 

Reagents	Final concentration	Volume
DMEM (1 g/L D-glucose, with GlutaMAX)	60% (v/v)	290 mL
Ham’s F12	40% (v/v)	190 mL
BioWhittaker antibiotic-antimycotic (100×)	1×	5 mL
Insulin-transferrin-selenium (100×)	0.5×	2.5 mL
2-Phospho-L-Ascorbate (100 mM)	0.5 mM	2.5 mL
Recombinant human EGF (10 mg/mL)	10 ng/mL	0.5 mL
Recombinant human PDGF-BB (10 mg/mL)	10 ng/mL	0.5 mL
Dexamethasone, USP micronized (10 mM)	10 nM	0.5 mL
Flexbumin (25%)	0.1% (v/v)	2 mL
Human serum (100%)	2% (v/v)	10 mL
*Notes:*
  - *All reagents used in this GMP culture formulation adhere to the GMP standards. During the development of the GMP-compliant formulation, we tested different GMP substitutes for the research-grade chemicals/reagents that are used in the lab-based culture medium [19]. This was done to ensure that the formulation meets the GMP standard. The corresponding reagents were comprehensively evaluated against the original research-grade components through rigorous cellular and molecular assays using at least three primary donor CSSC batches. After evaluating multiple GMP-grade reagents, we meticulously developed an optimized formulation that closely mirrors the research-grade culture medium.*
  - *It is worth noting that human serum can be lipid- and protein-rich, depending on the donors. This can cause the culture medium to appear cloudy with the presence of insolubilities, although it is sterile. For this reason, we suggest that the final medium should be filter-sterilized before use.*

**Laboratory supplies**
1. Cell strainer (40 and 70 μm pore size) (Fisher Scientific, catalog numbers: 22362537 and 22362548)
2. Cryovials (1.8 mL volume) (Fisher Scientific, catalog number: 1050026)
3. Tissue culture flask (TCF) 25 cm^2^ (Corning, catalog number: 430168), 75 cm^2^ (Genesse Scientific, GenClone, catalog number: 25-209); tissue culture dish (TCD) 30 mm (Falcon, catalog number: 353001), 60 mm (Falcon, catalog number: 353002), 100 mm (diameter) (Genesse Scientific, GenClone, catalog number: 25-202); tissue culture plate (TCP) 6-well (Fisher Scientific, catalog number: EB012927)
4. Sterile syringe (10 mL volume) (BD, catalog number: 305482)
5. Sterile centrifuge tubes (15 mL and 50 mL volume) (VWR, catalog numbers: 525-1069 and 525-1014)
6. Sterile microcentrifuge tubes (1.5 mL and 2 mL volume) (Fisher Scientific, catalog numbers: 05-408-129 and 02-681-321)
7. Sterile syringe filters (0.22 mm pore size) (Nalgene, catalog number: 726-2520)

## Equipment

1. Biological safety cabinet (Class II type A2/B2) (Baker, model: SteriGARD III Advance)
2. Phase-contrast microscope with 4×, 10×, and 20× objectives (Invitrogen, model: EVOS XL Core)
3. Stereomicroscope for dissection (0.8 to 16×) (AMScope, model: SM-1 LED 1445)
4. Humidified CO_2_ incubator (Thermo, model: HERAcell 150i)
5. Temperature-controlled benchtop centrifuge (Thermo, catalog number: 75004381)
6. Automated cell counter (Invitrogen, model: Countess^TM^)
7. Colibri toothed forceps
8. Vannas curved scissors (6 mm blade) for corneal procedures
9. Scalpel handle #3
10. Sterile surgical blade #10 (Integra Miltex carbon steel sterile surgical blades, model: 4-110)
11. Germinator glass bead sterilizer (Fisher Scientific, model: Microbead Sterilizer B1305-FIS)
12. Rotator (set at 30 rounds per minute, rpm)
13. Mr. Frosty^TM^ freezing container (with isopropanol) (Nalgene Cryo freezing container, model: 5100-0001)
14. Liquid nitrogen cell storage (Thermo, model: Thermolyne CY509975)
15. Digital balance (Denver Inst Co., model: TR2102)

## Procedure

- **Donor corneas**
**Donor selection:** Donor assessment will be performed by a certified eye bank (Eversight Eye Bank, Cleveland, OH, Chicago, IL, Ann Arbor, MI, and Clark, NJ). To be considered for donation, donors should be younger than 60 years old, with no history of cancer or drug use. Informed consent from the next of kin of all deceased donors is necessary. The corneas must be suitable for clinical use and free of any known disease, injury, or inflammation. Additionally, the donor endothelial cell count should be greater than 2,000 cells/mm^2^. Negative serology results for transmissible diseases (including but not limited to human immunodeficiency virus, hepatitis B and C, and syphilis) are also required. The time from death to tissue recovery and preservation must be less than 12 h. The time from preservation to arrival at the cell facility should not exceed 5 days.
*Note: It is important that one of the donor inclusion criteria is that the corneal endothelial cell count should be greater than 2,000 cells/mm^2^. A low corneal endothelial cell count may be associated with stromal edema and an altered stromal environment. This could affect the viability and cell features of native stromal cells, including possibly the CSSCs at the limbal stroma.*
**Cornea harvest and delivery:** Donor cornea harvest will be performed under sterile conditions by a trained technician from a certified eye bank. Corneas are preserved in Optisol GS (Bausch & Lomb) or in other equivalent FDA-approved storage solutions under hypothermic storage (2–8 °C) conditions, without freezing, for shipment to the cell facility. For shipment, each cornea is placed in a primary container (sterile moist chamber) (Figure 1A), then in a temperature-controlled shipping box (at 2–8 °C).
**Receiving corneal tissue:** On the arrival of shipment, confirm that the primary moist chamber is without cracks or leakage. Before opening, wipe clean the container with 70% alcohol.
**Corneal tissue sterilization:** Using sterile forceps from the Germinator glass bead sterilizer, and after cooling, transfer corneal tissue to a TCD60 with 6–8 mL of DMEM/F12 containing 1× antibiotic/antimycotic at room temperature and rinse two times for 10 min each (Figure 1B).
  **Figure 1.**
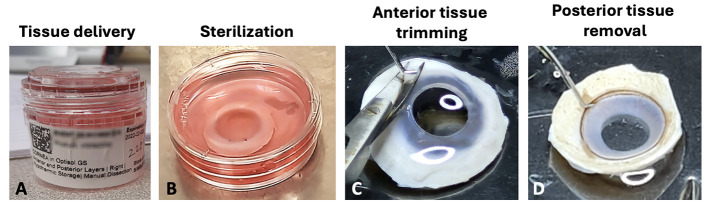
Processing steps of donor corneal tissue. (A) Donor corneal tissue stored in a primary container (sterile moist chamber) for delivery. (B) Tissue sterilization in DMEM with 2% antibiotics. (C) Removal of conjunctiva/tenons on the anterior side of donor cornea. (D) Clearing of all tissues on the posterior side.
- **Preparation prior to cell isolation**
**Collagenase NB6 working solution:** Thaw collagenase aliquot at room temperature for 10–15 min and add 1.35 mL of DMEM/F12 with 0.1% Flexbumin and 1× antibiotics/antimycotic (at room temperature) to make a working concentration of 1 mg/mL.
*Note: Avoid repeat freeze-thaw cycles of collagenase, as this process damages the physical protein structure and affects the enzymatic activities.*
**Fibronectin coating of culture surface:** Dilute GMP fibronectin at 500 mg/mL stock to 10 mg/mL working concentration. Apply 1 mL per TCF25 and coat at room temperature for 15–30 min. Remove solution, and the surface is ready for cell seeding.
- **Corneal tissue processing**
  1. Prepare 4–5 TCD100 with sterile PBS (up to 15 mL). Transfer corneal tissue to the first TCD100. Under stereomicroscopy magnification 0.8–2×, remove conjunctiva/tenons on the anterior side with a pair of toothed forceps and curved Vannas micro-scissors (Figure 1C).
*Note: Ensure the complete removal of conjunctiva and tenons to avoid contaminating fibroblasts in the CSSC culture. This step can be assisted by further scraping the anterior surface using a #10 surgical blade.*
  2. Using a #10 surgical blade, scrape to remove corneal and conjunctival epithelium.
*Note: A complete removal of epithelium is necessary to avoid epithelial cells overgrowing the CSSC in culture. The use of dispase can be included but no GMP reagent is available.*
  3. On the posterior side, clear all tissues (including iris, corneal endothelium, and trabecular meshwork tissues) with a #10 surgical blade (Figure 1D).
  4. After rinsing the corneal tissue in the second TCD100 with sterile PBS (up to 15 mL), position the corneal tissue with the posterior side facing up. Remove the central cornea using a circular trephine (8 mm diameter), leaving the peripheral cornea (approximately 0.5–1 mm width) and the limbus (a transition from the transparent cornea to the opaque scleral tissue) undisturbed (Figures 2A–C).
    **Figure 2.**
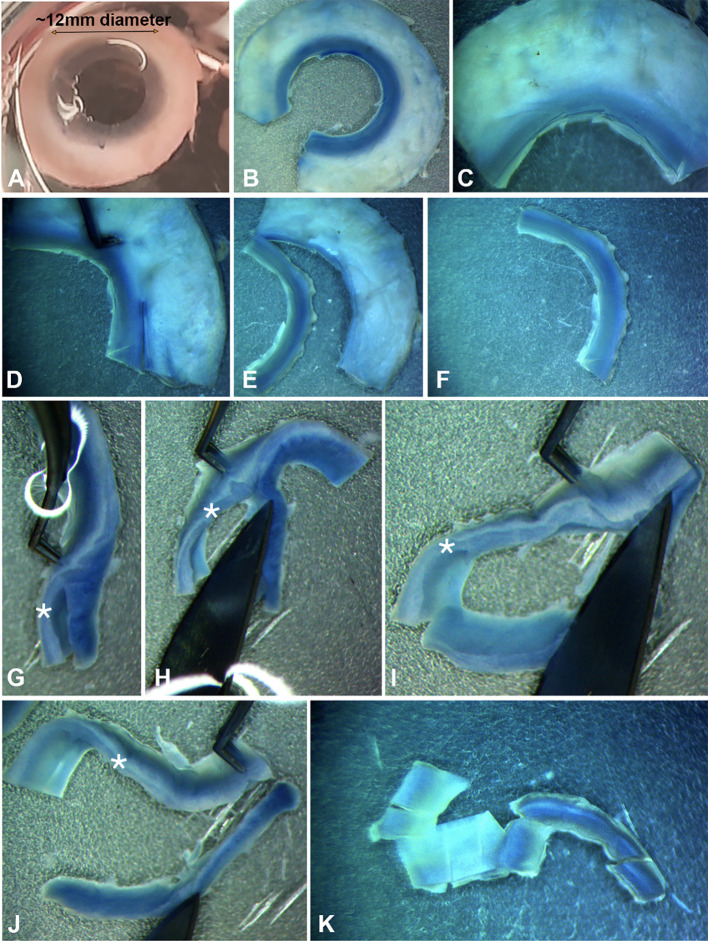
Steps of isolating anterior limbal stroma. (A) Whole adult cornea (~12 mm diameter) without the central region after removal by a circular surgical trephine. (B–C) Cut the corneal rim into quadrants. (D–F) Trimming of limbal strip with ~0.5 mm on scleral side and 1–1.5 mm on corneal side. (G–I) Making vertical cuts to separate anterior limbal stroma from the rest of tissue. (J) Separation of anterior limbal stroma. (K) Cuts of anterior limbal stromal strip into small blocks for efficient collagenase digestion. *denotes the anterior limbal stroma.
- **Anterior limbal stroma isolation**
  1. Cut the corneal rim into quadrants with a #10 surgical blade.
  2. At the exterior side of the limbus, make a vertical cut to remove the sclera, leaving approximately 0.5 mm (width) scleral tissue with the limbus (Figures 2D–F).
  3. After rinsing the limbal strip quadrants in the third TCD100 with sterile PBS, position the limbal strip slightly vertical and trim to obtain the anterior 1/3 to 1/2 of the thickness (Figures 2G–J).
*Note: CSSCs are located at the anterior limbal stromal region near the basement membrane [18,20].*
  4. After rinsing the anterior limbal tissue in the fourth TCD100 with sterile PBS, cut the tissue into small blocks, approximately 1 mm^3^ in size (Figure 2K).
  5. Transfer tissue blocks to the 2 mL tube with 1 mg/mL collagenase NB6 solution (see Recipes).
  6. Digest tissues for 6–8 h at 37 °C with slow rotation at 30 rpm.
*Note: At the last hour of digestion, triturate the digest with a P1000 pipette tip 5–10 times to assist cell dislodging from the loosened stromal matrix by collagenase activity. Return the mixture to 37 °C incubation for the last hour of digestion.*
  7. After digestion, triturate the lysate with a P1000 or P200 (depending on the extent of tissue digestion).
  8. Pass the digest through a 70 μm cell strainer to obtain a single-cell suspension, wash the cell strainer with 10 mL of PBS, and collect into a 50 mL centrifuge tube.
  9. Centrifuge at 250× *g* in a swinging-bucket rotor for 5 min at room temperature.
  10. Gently resuspend the pellet in 3 mL of GMP SCCM (see Recipes) at room temperature and transfer to a 15 mL centrifuge tube.
*Note: The pellet should not be visible, as not many cells can be obtained from a single anterior limbal stroma. If a visible pellet is noticed, it could mean that the limbal stromal matrix has not been digested properly. Check that the cell strainer is being used correctly and repeat the collagenase digestion for 1–2 h followed by trituration to ensure that cells are well dislodged. Then, repeat the filtration process through the cell strainer to obtain a single-cell suspension.*
  11. Spin again at 250× *g* in a swinging-bucket rotor for 5 min at room temperature.
  12. Resuspend the pellet in GMP CSSC (5 mL of volume) and seed to the fibronectin pre-coated TCF25.
  13. Place the primary culture (passage 0, P0) in a humidified CO_2_ incubator at 37 °C with 5% CO_2_ balanced with air.
- **Primary donor CSSC culture at P0**
  Under phase-contrast microscopy, check the primary cell clusters (with 4–10 cells) that usually appear within 3–7 days (Figure 3). They should be homogeneously small-sized cells. Mark 2–3 clusters that appear early in the P0 culture and monitor their confluence status. Once sufficient CSSC clusters are detected, remove the culture medium and replenish with fresh GMP SCCM. Repeat medium change every 48–72 h.
  The clusters expand with increasing cell numbers and become confluent at the center region (Figure 3). When those marked clusters reach 50%–70% confluence and with increasing cell–cell contacts, the P0 culture should be sub-passaged by trypsinization. If no clusters appear after 14 days, the primary culture is considered to have failed and should be discarded.
*Notes:*
  - *Our GMP medium formulation has been developed based on research published earlier [18,10] to support the growth of donor CSSCs. Although other cell types such as endothelial cells and neutrophils may briefly attach to the culture surface, they do not proliferate or form detectable colonies.*
  - *On days 3–4, if no cells adhere to the culture surface, add 1–2 mL of fresh medium without discarding the used medium to replenish the nutrients and growth factors. Such supplements can improve cell viability and attachment.*
  - *Multiple CSSC clusters/clones can be generated during P0 culture, and they have different initiation times, cell density, morphology, homogeneity, and growth rate. Early-formed clones tend to contain more cells and be more confluent than late-formed clones. To monitor cell growth and avoid over-confluence in early clones, it is recommended to mark the first few clones that appear in the early stages of P0 culture. Once these marked colonies reach approximately 50%–70% confluence or increased cell–cell contact and packing, it is necessary to sub-passage the culture. This will prevent stem cell changes that may result from high confluence, e.g., cell differentiation and the loss of stem cell properties.*
  - *The quality and yield of primary CSSCs can be affected by various factors. These factors include the freshness and viability of the donor corneal tissue, sub-optimal conditions during tissue processing, and poor cell survival or cell damage after the tissue’s enzymatic digestion. Additionally, the transition from an in vivo matrix environment to an in vitro condition with different substrate adhesion can be a crucial factor in determining cell survival and behavior. Therefore, it is not uncommon to have no viable CSSCs in the P0 culture. Even though the early clusters can form and be detected, this does not guarantee the continuous survival and growth of primary cells. In fact, primary cells may undergo early senescence or cell death due to poor adaptability to the new environment.*
  **Figure 3.**
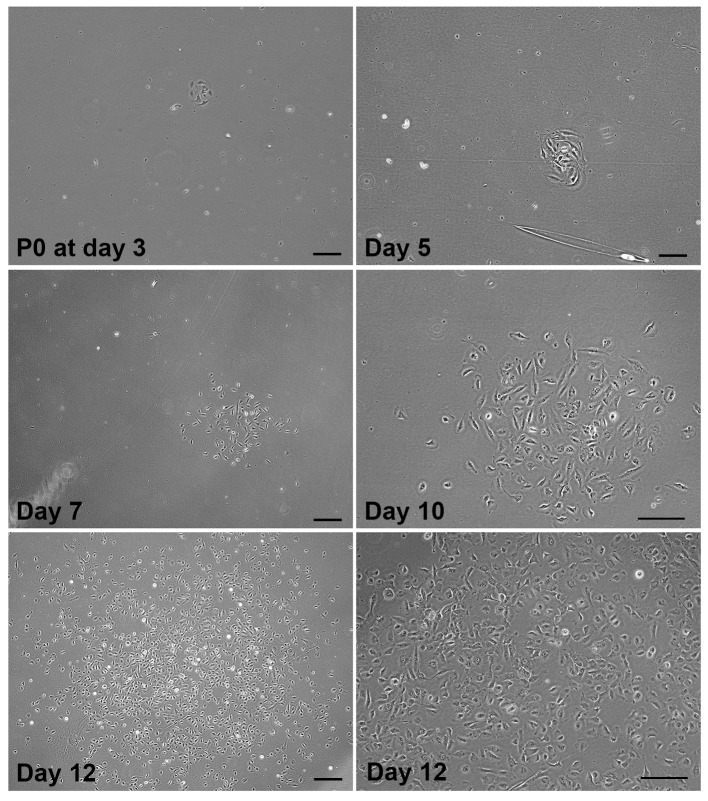
Primary good manufacturing practice (GMP)-corneal stromal stem cell (CSSC) cluster (TPF-23-29) at P0 and cell propagation. Phase-contrast micrographs showing the cell cluster with 6–8 cells detected at day 3 post-seeding of primary CSSCs freshly isolated from anterior limbal stroma. The expansion of the same cluster at day 5, 7, and 10 showed an exponential increase in stem cell number. At day 12, the clonal expansion generated colonies with >500 cells. Magnified image at day 12 (bottom right) shows cells with moderate cell–cell contact at approximately 70% confluence. This stage is suitable for sub-passaging and cryopreservation. Scale bar, 50 μm.
- **P0 sub-passaging, viable cell count, and cryopreservation**
  When early cell clusters at P0 reach 50%–70% confluence with increasing cell–cell contacts (Figure 3), cells are sub-passaged by trypsinization:
  1. Remove the medium by aspiration.
  2. Add 5 mL of sterile PBS to each TCF25, rinse, and discard.
  3. Add 1 mL of TrypLE Select CTS and allow brief trypsinization for approximately 1 min at 37 °C.
  4. Dislodge cells from the culture surface by rocking the flask side to side or pipetting 5–10 times.
  5. Collect cell suspension into a 15 mL conical centrifuge tube. Rinse TCF25 with 5–8 mL of sterile PBS and collect to the cell suspension.
  6. Centrifuge at 250× *g* for 5 min at room temperature in a swinging-bucket rotor.
  7. Resuspend the cell pellet in GMP SCCM (1 mL) and mix well to single-cell suspension.
  8. Mix 10 mL of cell suspension with 10 mL of 0.4% Trypan blue for cell counting and viability check.
  9. Calculate total cell yield. Take 2,000 cells for P1 culture in a new TCF75 pre-coated with GMP fibronectin (see Note). Add 10–12 mL of GMP SCCM to each TCF75. For even seeding, gently rock the flask from side to side. Incubate the flask at 37 °C with 5% CO_2_. Refresh medium every 2–3 days. Examine cell distribution and growth confluence under the phase-contrast microscope.
  10. Calculate the number of P0 cells and number of aliquots for cryopreservation (see Note).
  11. Centrifuge the cell suspension at 250× *g* for 5 min at room temperature in a swinging-bucket rotor.
  12. Prepare cryovials (n = 6 each cell batch) labeled with cell ID, quantity, and date of preparation.
  13. Prepare cryopreservation medium by diluting CS10 with GMP SCCM (1:1 vol/vol) to CryoStor CS5 (see Recipes). Add 3 mL of cryopreservation medium to resuspend the cell pellet.
  14. Put 0.5 mL of cell suspension to each labeled cryovial.
  15. Freeze cryovials in a controlled freezing apparatus at -80 °C from overnight to 24 h.
  16. Transfer frozen vials to liquid N_2_ and store at the gas phase position.
*Notes:*
  - *The cell seeding density is suggested after our evaluation with more than 15 primary CSSC batches. The cells propagated with the GMP SCCM for 5–7 days in a TCF75 without reaching more than 80% confluence. However, this may vary from case to case. Some stem cell batches may grow faster and others not. Hence, it warrants an optimization based on the suggested seeding density.*
  - *In Funderburgh et al. [20], authors showed that healthy primary CSSCs have 50–70 doublings before undergoing senescence, apoptotic cell death, or adverse cell features [20]. This represents that CSSCs can generally be sub-passaged up to three times to Passage 3 without detectable cell changes.*
  - *It is advisable to freeze P1 cells at 2,000–6,000 cells/cryovial so that the cells after thawing can be seeded up to 3× TCF75 to generate increasing cell quantity for P3 experiments.*
- **Thawing CSSCs**
  When thawing CSSCs, allow at least one passage of recovery and expansion before plating for experiments. Since the cells should be used within the first three passages, it is advisable to freeze more cell aliquots at P1 so that the thawed P2 cells can be recovered and expanded for p3 experiments.
  1. Prepare fibronectin-coated culture surface (see Recipe 5).
  2. Per cryovial, prepare a 15 mL conical centrifuge tube with 9 mL of GMP SCCM.
  3. Remove the cryovial from liquid N_2_ storage and float it in a 37 °C water bath until a tiny ice clump is left.
  4. Transfer 1 mL of GMP culture medium to the cryovial, mix by pipetting up and down, and transfer the cell suspension to the prepared 15 mL tube with culture medium (10 mL) at room temperature.
  5. Mix well and centrifuge the tube at 250× *g* for 5 min at room temperature.
  6. Remove supernatant and add 1 mL per TCF75 (~2,000 cells per mL) to resuspend the pellet by carefully pipetting up and down under single-cell suspension.
  7. Add 10–12 mL of GMP SCCM to each fibronectin-coated TCF75 and transfer cell suspension at 1 mL per TCF75.
  8. To evenly distribute the cells, gently rock the flask from side to side.
  9. Put the flasks inside the CO_2_ incubator at 37 °C.
  10. Examine cell growth and distribution under phase-contrast microscopy the next day. After having visible cell colonies, perform GMP medium change every other day.
*Notes:*
  - *Good CSSC will give clonal expansion from single cells in 3–5 days.*
  - *During propagation, stem cell differentiation from CSSCs to corneal stromal keratocytes, the expression of stem cell markers [e.g., ABCG2 (ATP-binding cassette super-family G member 2), Pax6, nestin, Bmi-1 (B lymphoma murine leukemia viral insertion region 1)], in vitro quality control, and in vivo efficacy tests in reducing corneal scar formation in animal models of corneal stromal injury can be executed. Details of these assays are given in our early studies [10,15,16,19].*

## Validation of protocol

This protocol has been used and validated in the following research article:

• Santra et al. [19]. Good manufacturing practice production of human corneal limbus-derived stromal stem cells and in vitro quality screening for therapeutic inhibition of corneal scarring. *Stem Cell Res Ther*. 2024 Jan 8;15(1):11. DOI: 10.1186/s13287-023-03626-8.

The GMP protocol is optimized from the previous donor CSSC protocol using research-grade chemicals and reagents. It was validated in the following publications:

1. Du et al. [18]. Stem Cells; DOI: 10.1634/stemcells.2004-0256
2. Du et al. [24]. Invest Ophthalmol Vis Sci; DOI: 10.1167/iovs.07-0587
3. Du et al. [25]. Stem Cells; DOI: 10.1002/stem.91
4. Basu et al. [10]. Sci Trans Med; DOI: 10.1126/scitranslmed.3009644
5. Hertsenberg et al. [12]. PLoS One; DOI: 10.1371/journal.pone.0171712
6. Shojaati et al. [26]. Stem Cells Trans Med; DOI: 10.1002/sctm.17-0258
7. Shojaati et al. [11]. Stem Cells Trans Med; DOI: 10.1002/sctm.18-0297
8. Khandaker et al. [15]. Exp Eye Res; DOI: 10.1016/j.exer.2020.108270
9. Weng et al. [14]. Eye Vis; DOI: 10.1186/s40662-020-00217-z
10. Jhanji et al. [16]. Int J Mol Sci; DOI: 10.3390/ijms23136980
11. Yam et al. [17]. J Adv Res; DOI: 10.1016/j.jare.2022.05.008

## General notes and troubleshooting

**General notes**

1. Primary CSSC colonies typically manifest within 3 to 5 days. Clonal growth on this selective medium is a unique characteristic of stem cells. Although other cell types, such as endothelial and neutrophils, may attach, they do not survive as colonies. CSSC clones, which have uniformly small-sized cells, can be identified visually.
2. If no colonies appear within 14 days, the culture is deemed poor or without growth, and it should be terminated by adding diluted bleach solution before disposal.
3. GMP stem cell culture medium (SCCM) can be stored at 4 °C for a maximum of one week.

**Troubleshooting**

**Figure 4.**
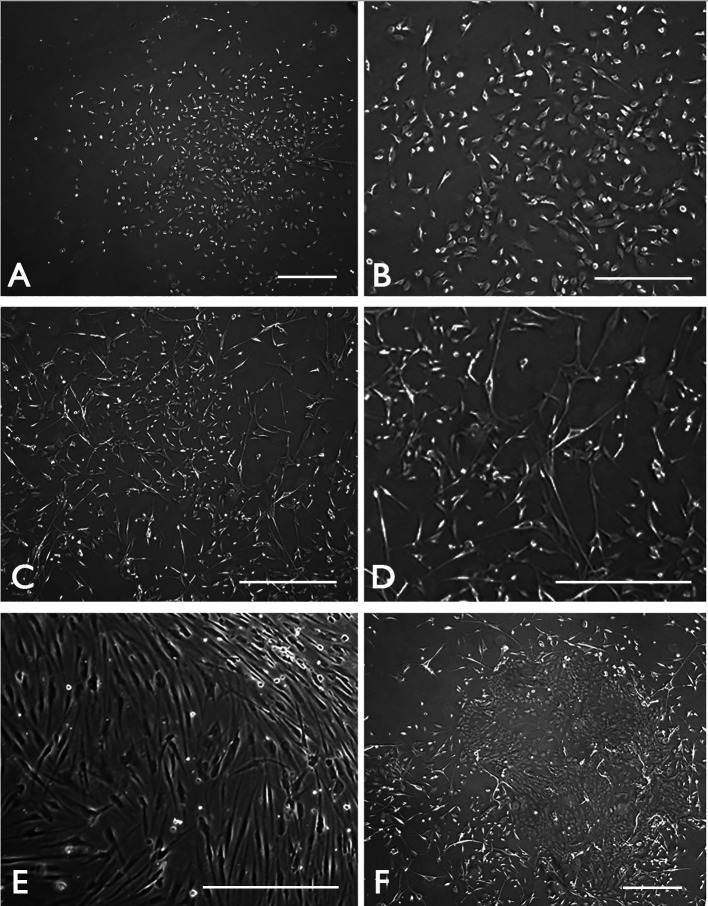
Potential contaminating cell types in primary good manufacturing practice (GMP)-corneal stromal stem cell (CSSC) culture. Phase-contrast micrographs showing (A) proper CSSC cluster with clonal expansion at P0 and (B) the growth of small-sized proliferating cells at P1. (C) Growth of fibroblast-like cells can occur in CSSC culture. (D) Under higher magnification, they display an heterogenous slender to stellate shape. (E) After sub-culture, the fibroblasts overgrow the CSSCs. (F) In case the limbal epithelium is not completely removed, epithelial colonies are formed due to the fast-growing limbal epithelial stem cells. Scale bar 120 μm.

Problem 1: A major challenge faced is the transition of CSSC to fibroblast types, particularly in serum and growth factor–supplemented conditions. The fibroblasts are different from the small-sized CSSCs and have a heterogenous slender to stellate shape. However, these fibroblast-like cells can arise within the CSSC clones (Figures 4C and D).

Solution: If there is mild fibroblast growth among CSSC clusters, perform a brief trypsinization using pre-warmed TrypLE Select for less than a minute’s incubation at 37 °C. This will dislodge the primary CSSCs, which can be collected for the next passaged culture. This will cause a substantial loss of CSSCs, but the cell purity/enrichment can be improved. If fibroblasts persist substantially, terminate the culture (Figure 4E). Additionally, the incomplete removal of limbal epithelium (refer to step C2) will result in the formation of epithelial cell colonies (Figure 4F). They usually grow faster than CSSCs, and the culture should be terminated.

Problem 2: The preservation of corneal tissue in Optisol GS has not been optimized for the maintenance of CSSC viability. Prolonged storage of donor tissues in Optisol may have unexpected adverse effects on CSSCs.

Solution: It is recommended to process the corneal tissues as soon as possible upon receiving the samples, to avoid any potential harm to CSSCs.
